# Engineering strategies towards overcoming bleeding and glial scar formation around neural probes

**DOI:** 10.1007/s00441-021-03567-9

**Published:** 2022-01-14

**Authors:** Elisabeth Otte, Andreas Vlachos, Maria Asplund

**Affiliations:** 1 Department of Microsystems Engineering (IMTEK), University of Freiburg, Georges-Köhler Allee 201, 79110 Freiburg, Germany; 2 Department of Neuroanatomy, Institute of Anatomy and Cell Biology, Faculty of Medicine, University of Freiburg, Freiburg, Germany; 3 Center BrainLinks-BrainTools, University of Freiburg, Freiburg, Germany; 4 Center for Basics in Neuromodulation (NeuroModulBasics), Faculty of Medicine, University of Freiburg, Freiburg, Germany; 5 Division of Nursing and Medical Technology, Luleå University of Technology, Luleå, Sweden; 6 Freiburg Institute for Advanced Studies (FRIAS), University of Freiburg, Freiburg, Germany

**Keywords:** Neural interfaces, Neural probes, Glial scarring, Electrophysiology, Bioelectronics, Microelectrode arrays

## Abstract

Neural probes are sophisticated electrophysiological tools used for intra-cortical recording and stimulation. These microelectrode arrays, designed to penetrate and interface the brain from within, contribute at the forefront of basic and clinical neuroscience. However, one of the challenges and currently most significant limitations is their ‘seamless’ long-term integration into the surrounding brain tissue. Following implantation, which is typically accompanied by bleeding, the tissue responds with a scarring process, resulting in a gliotic region closest to the probe. This glial scarring is often associated with neuroinflammation, neurodegeneration, and a leaky blood–brain interface (BBI). The engineering progress on minimizing this reaction in the form of improved materials, microfabrication, and surgical techniques is summarized in this review. As research over the past decade has progressed towards a more detailed understanding of the nature of this biological response, it is time to pose the question: Are penetrating probes completely free from glial scarring at all possible?

## Introduction

Rapid technological development continuously provides new techniques for the study of brain signals. Of particular importance are implantable microelectrode arrays, called neural probes. Neural probes allow electrophysiological signals to be recorded from within the brain down to the level of individual neurons (Buzsaki 2004). Furthermore, electrical stimulation from such electrode arrays can provide therapeutic uses to modulate the neuronal activity or even restore sensory function, such as vision using a direct cortical interface (Normann and Fernandez 2016; Chen et al. 2020). Despite the vast technological advancement, only a handful of systems have received FDA approval for clinical applications to date (Obidin et al. 2020).

A fundamental shortcoming of all invasive probes is perturbation of the targeted neural networks. First, the act of implantation induces trauma when the probe cuts through blood vessels and neural connections as it proceeds into deeper structures, which in turn triggers an acute inflammatory reaction (Polikov et al. 2005; Tresco and Winslow 2011; Kozai et al. 2012). Second, the presence of the probe triggers a continued foreign body response represented by glial scarring, chronic inflammation, and loss of nearby neurons (Biran et al. 2005; McConnell et al. 2009). Third, additional mechanical trauma might occur as mechanically incompatible probes move relative to the tissue (Harris et al. 2011). Consequently, the tissue surrounding the probe experiences changes in the initial weeks post-implantation, which commonly results in reduced recording signal quality (Kozai et al. 2015; Nolta et al. 2015) and may increase the stimulation thresholds required to evoke a specific response. If mechanical failure of the implant can be excluded, the reduced signal quality is related to a reduction in neuronal activity surrounding the device. An increased distance between the electrode and viable neurons, as well as the insulating properties of the glial scar itself, are obstructing efficient signal transduction between neurons and electrodes (Barrese et al. 2013; Wellman et al. 2018; Usoro et al. 2021). Notably, other studies have shown that histological evidence of gliosis and loss of neurons do not always correlate with poor signal quality and, conversely, the absence of such findings does not guarantee a functional neural interface (Purcell et al. 2009; Kozai et al. 2014; McCreery et al. 2016; Michelson et al. 2018).

Research in recent decades has significantly advanced our understanding of how gliosis and associated inflammation around the implanted probes result in performance decline over time. We now understand that surgical skill, probe biomechanics, tissue-surface interaction, and the cross-sectional footprint are critical in reducing gliosis and inflammation. However, the interplay of these variables remains unclear, and we must identify technological solutions that provide the most influence over the outcome. Typically, a trade-off is necessary between functions, such as the total number of electrodes and electrode integration density, implantability, and longevity (Wellman et al. 2018).

It is somewhat problematic that gliosis around devices has previously been viewed as one reaction (foreign body response), as selecting improved strategies to mitigate this response requires a detailed view of multiple potential underlying causes. Stiff probes generally cause minimal insertion trauma, while flexible devices rely on special tools (insertion shuttles). Moreover, an uncomplicated initial surgery may be decisive to reduce the extent of inflammation in the first few weeks (Kozai and Kipke 2009; Kozai et al. 2010) and preserve more neurons adjacent to the device (Wellman et al. 2018). Bleeding during surgery will result in blood deposits within the brain, which by itself may be problematic (Schachtrup et al. 2010) even though for implantable applications, in tissues other than the brain, surface adsorption of blood proteins is not always viewed negatively. Fibrin deposition, for example, may act as a scaffold for tissue regeneration, helping bridge the transition from biotic to abiotic material (Wu et al. 2020). However, blood proteins in the brain may trigger gliosis and seizure activity and result in continued leakage over the blood–brain interface (BBI), compounding the blood deposit problem (Shimon et al. 2015). Reducing the presence of blood deposits, particularly preventing adsorption onto the surface of the probe, could therefore be a target for improved surface biocompatibility.

Independent of these initial reactions, micro-movements between probe and tissue can by themselves drive gliosis. Naturally occurring brain pulsations due to respiratory cycles and cardiac activity result in relative movements of the probe if it is rigidly fixed or tethered to the skull (Gilletti and Muthuswamy 2006; Karumbaiah et al. 2013; Prodanov and Delbeke 2016). Various methods have been proposed to compensate for this effect, such as using a cardiopulmonary bypass (Britt and Rossi 1982) or micro-actuated probes that can be repositioned to compensate for brain movement (Muthuswamy et al. 2005). However, these methods fail to address the larger incompatibilities in mechanical properties between the probe and brain tissue. Additionally, brain movements may result in a cutting motion and thereby secondary trauma to the surrounding tissue. Probes made from flexible materials offer a practical solution to this problem and have shown promise for sustaining a nearly gliosis-free contact (Subbaroyan et al. 2005; Sohal et al. 2014; Sridharan et al. 2015; Boehler et al. 2017; Luan et al. 2017; Chung et al. 2019; Song et al. 2020).

Finally, the cross-section of the probe should be considered, a quality that influences all the described injury mechanisms. Stiff but slender devices may demonstrate remarkable tissue integration (Stice et al. 2007), which is also the case for silicon structures, despite their rigid mechanical properties (Seymour and Kipke 2007). Outstanding recording stability was achieved by combining a flexible device with a small cross-section (Luan et al. 2017; Zhou et al. 2017). There are several possible explanations for why a small cross-section alone may allow nearly seamless tissue integration not permitted by a larger device. A thin probe will be more flexible than a thicker probe and will displace less tissue upon implantation. Probes with small cross-sections allow for reduced adsorption of biomolecules, potentially triggering gliosis (Schachtrup et al. 2010). In addition, as all neurons critically depend on oxygen supply, neuronal health relies on proximity to capillaries. From the neuron’s perspective, a larger device placed in the tissue constitutes a wall, restricting oxygenation. Thus, the smaller the probe, the less impact it will have on the natural perfusion of the tissue, and the more likely the surrounding neurons will remain healthy (Karumbaiah et al. 2013). Researchers have determined that even in the presence of blood vessels, reduced perfusion surrounding neural probes impacts neuronal health and activity (Kozai et al. 2012; Michelson et al. 2018; Welle et al. 2020). In summary, making probes smaller may be more critical to their success than making them flexible (Lee et al. 2017; Kozai 2018).

There are many parameters to consider when designing “seamless” neuro-technological devices, and these parameters interact in a complex manner and collectively contribute to the foreign body response. The aim of this review is twofold: (1) to provide an overview of important design parameters, which can serve as a reference for probe engineers in their choice of material, dimensions, and overall interface technology, and (2) to serve as an introduction for scientists from other disciplines who may contribute new perspectives on the causes of reactive gliosis and most importantly, new discussions regarding how to address this problem best.

## A brief history of intracortical neural interfaces

Microfabricated neural probes were first developed in the late 1960s, although the broad adoption of these neuroscience tools did not occur until decades later (Wise et al. 1970; Buzsaki 2004). The longstanding, typical device for intracortical electrophysiology was a micro-wire consisting of an insulating shell and a metal core. In this case, the exposed metal at the tip comprises the microelectrode. As the fabrication of such electrodes did not require any sophisticated equipment or specific expertise, they were a relatively cheap and accessible solution to be manufactured directly in the neuroscience laboratory. With the evolvement of tetrodes (a bundle of four closely packed microwires), triangulation of signals could be applied to identify single neurons in a complex signaling environment (Harris et al. 2000; Rossant et al. 2016). This advance increased the interest in the more sophisticated multielectrode configurations enabled by appropriate micro-manufacturing methods. After the initial conceptual publications (Wise et al. 1970), an expansive phase for microelectrochemical systems (MEMS)-based neurotechnology followed. Today, over 50 years later, MEMS-probes have widely replaced the more simplistic microwire solutions. The recent development of complementary metal–oxide–semiconductor (CMOS) probes has allowed the number of individual electrodes per device to increase from less than 50 to hundreds, and in exceptional cases, even permitting thousands of recording sites on a single device (Jun et al. 2017; Raducanu et al. 2017; Dorigo et al. 2018; Angotzi et al. 2019; Steinmetz et al. 2021). Improved technology allows the brain to be studied at a more refined resolution, over extended time, and using more informative and complex behavioral models. The advancement of neurotechnological methods thereby contributes to the forefront of neuroscience.

MEMS-based neurotechnological devices have exceptional potential to enable future “neuroprosthetic” or “bionic” therapies (Lawand et al. 2011; Normann and Fernandez 2016). Notwithstanding, requirements naturally differ between neurotechnology developed for neuroscience, pre-clinical neuroscience, or clinical applications (Stieglitz 2020). However, as the process technology and design concepts are widely the same, these three application fields are not further differentiated in this review. Retina implants and brain-machine interfaces based on the Utah Electrode Array (see next section) are examples of early progress towards MEMS-based neurotechnology in humans (Simeral et al. 2011; Mills et al. 2017). Nevertheless, only recently have these technologies reached the maturity needed for clinical adoption and promoted broader industrial interest and investment.

## Design aspects of intracortical probes

### Probe types

**Fig. 1:**
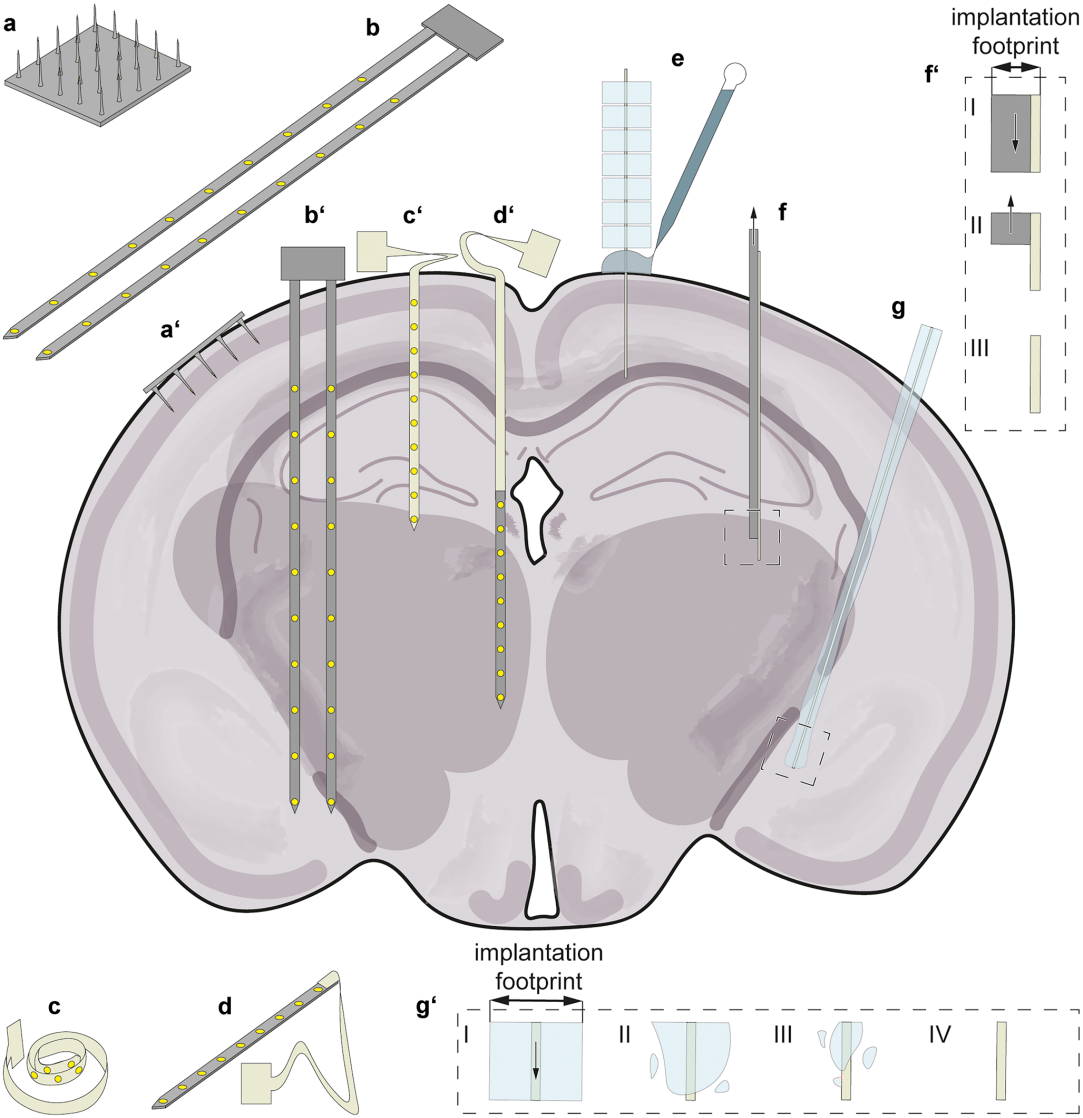
Illustration of different probe categories (left) and implantation methods for flexible probes (right): (**a**) Utah-probe with electrodes (**a**’) implanted in one cortical layer; (**b**) Michigan-probe with electrodes (**b**’) distributed amongst multiple depths after implantation; (**c**) flexible probe depicted in the Michigan-probe style (**c**’) implanted; (**d**) hybrid probe consisting of a stiff tip and flexible base and shank section (**d**’) floating after implantation; (**e**) stepwise implantation of a flexible probe by piecewise dissolving of the stiffening material outside of the brain; (**f**) implantation of a flexible probe using a shuttle with the detailed view (**f**’): (**f**’-I) the flexible probe is assembled to the shuttle and implanted into the desired position. (**f**’-II) The shuttle is retracted from the brain (**f**’-III) leaving only the flexible probe behind. (**g**) Implantation of a coated flexible probe with the detailed view (**g**’): (**g**’-I) the flexible probe is coated with a bioresorbable material and implanted into the desired position. (**g**’-II) and (**g**’-III) after implantation, the coating is gradually dissolved until only the flexible probe is left behind (**g**’-IV). The implantation footprint is larger than the probe cross-section in (**f**) and (**g**), as shown in insets (**f**’) and (**g**’)

As neural microelectrode arrays, or probes, may serve for a diverse set of experiments and applications, there is no unified view of what signifies an excellent neural probe. As a consequence, a variety of technologies are pursued in parallel to meet the shifting requirements. A graphical overview of the most common ones is provided in Fig. 1a–d. A standard categorization is to distinguish between electrode arrays that are bed-of-needle arrays (Fig. 1a) and shanks (Fig. 1b). Shank-based probes are commonly identified as Michigan-style probes, referring to the early work pursued by Wise and colleagues at the University of Michigan, and can be further divided into single- and multi-shank configurations (Wise et al. 1970). The electrodes are placed in one or several rows along the penetraing shank, which is useful for interfacing with several cortical layers in parallel (Fig. 1b’). Typical shanks only have microelectrodes on the front side with the rear side being completely insulated and passivated. This one-sidedness is a simple consequence of the planar wafer-level microfabrication in which the probe is an accumulation of layers. Every layer is patterned using photolithography and etching from the front side. Multi-sided fabrication processes are also possible, although practically more challenging to complete (Shin et al. 2017).

The other main electrode array category would be the bed-of-needle, commonly referred to as Utah-style probes, because the Utah Electrode Array has been most prominently representing them (Campbell et al. 1991; Rousche and Normann 1998). The electrodes of a bed-of-needle array, as depicted in Fig. 1a, are on the tip of each “needle” and could either compose a monolithic MEMS structure or be an assembly of many microwires (Patel et al. 2016). Thus, the bed-of-needle array makes it possible to interface many locations at the same cortical depth (e.g., all electrodes within the same cortical layer) as shown in Fig. 1a’. Hybrids between shanks and bed-of-needle arrangements are also possible, such as multi-shank probes stacked into a bed-of-needle array, which would allow the interfacing of multiple layers and coverage of several locations within each layer.

A relatively recent addition to these concepts are nanomeshes, or brush-like probes, described as multielectrode versions of ultra-thin wire electrodes or, in rare cases, even tissue-engineered single axons (Zhou et al. 2017; Dai et al. 2018; Serruya et al. 2018; Guan et al. 2019). Typically, these mesh- or brush-like designs would have a much smaller cross-section than a shank. Consequently, few electrodes per individual fiber are compensated by having more fibers interpenetrating the brain. It is generally difficult to draw sharp boundaries between these categories of probes, as a brush may appear to be a multi-shank or bed-of-needle style device but with a thinner and less rigid structure.

### Probe-tissue biomechanics

Another important distinction is that between stiff (typically silicon-based; Fig. 1a and b) and flexible (typically polymer-based; Fig. 1c) devices. This differentiation affects not only the long-term tissue integration but also the fabrication and implantation techniques (Fig. 1c’ and e–g). In principle, the terminology (shank vs. bed-of-needle) outlined above applies regardless of whether the device consists of stiff (rigid) or flexible construction materials. It mainly refers to the arrangement of the electrodes, not to the construction material itself. Flexible devices offer additional versatility as a planar device can be folded (Kim et al. 2018) or twisted into a three-dimensional construct. Dedicated substrate materials can even offer the possibility of softening post-implantation (Ware et al. 2014). The term “soft” is sometimes used to describe probes made from non-rigid materials, although it should be noted that soft and flexible are not synonymous. Softness is a surface characteristic, while flexibility is a characteristic of the bulk material. A silicon probe coated with a hydrogel becomes soft but is not flexible. An extremely thinned but uncoated silicon probe can become flexible but is not soft. A standard polymer probe, however, might be soft and flexible at the same time. The boundary between flexible or rigid refers to the construction material and, in addition, depends on the geometry of the complete device. Specifically, the Young’s modulus *E* is only one variable in the beam equation (Eq. 1) in addition to the width *w* and thickness *t* of the device (Timoshenko and Gere 1988).

1 $$K=E\frac{w{t}^{3}}{12}$$

In other words the bending stiffness *K* depends on both the substrate material and the probe geometry, and  thinning the probe to decrease its thickness *t* will have a more significant influence on *K* as reducing the probe width *w*. Recent work providing flexible probes based on extremely thin silicon (Wellman et al. 2019; Otte et al. 2020), or flexible brush-like arrays based on carbon fibers (Guitchounts and Cox 2020) emphasizes this point.

Hybrid probes (as illustrated in Fig. 1d) provide the advantages of combining stiff and flexible parts (Kim et al. 2014; Xue et al. 2018; Barz et al. 2020; Novais et al. 2021). Barz et al. (2020) presented this type of hybrid probe with a compact silicon-based tip section consisting of densely packed electrodes to be addressed by an on-chip multiplexer. In contrast to conventional Michigan probes, in this case, the PI ribbon cable is partly implanted, resulting in the flexible elongation of the silicon shank and achieving a mechanically mainly decoupled, floating CMOS-probe (Fig. 1d and d’) (Barz et al. 2020). With the trend toward mechanically decoupled floating probes, combinations of flexible and rigid materials are becoming increasingly common in implantation setups. For example, another variant combines several flexible Michigan-style probes via CMOS chips in a single head stage (Chung et al. 2019). Whether such a setup may be considered a hybrid probe is a matter of definition.

### Does the optimal probe exist?

There is currently no consensus that one type of device is superior to all others, as the best choice of technology depends on the requirements and priorities of a specific application. Presently, none of the existing technology fully addresses high-resolution interfacing (determined by integration density of electrodes), coverage (the ability to interface multiple brain regions), and long-term stability of the interface’s technological and biological components. Mechanical flexibility and small cross-section are key components for long-term stability. The latter is the main driving force for the recent evolution of brush- and mesh-style arrays (Karumbaiah et al. 2013; Dai et al. 2018; Guan et al. 2019). Nevertheless, it remains challenging to integrate the desirable electrode numbers (hundreds to thousands) on exceedingly small devices. Previously, electrode miniaturization was a limiting factor (Boehler et al. 2020). Recent advances in materials allow astonishing miniaturization of electrodes, maintaining low impedances and high charge injection capacities (Boehler et al. 2015; Boehler et al. 2020). However, as the feedlines and insulation will add to the total dimensions of the device, the achievable resolutions in the fabrication processes (thickness of insulation, width of connection lines) will limit miniaturization and enforce a trade-off between electrode numbers and device cross-section.

Miniaturization of shanks comes with various challenges depending on the fabrication method, which typically differs between flexible and rigid shafts. While the realization of probes on flexible substrates generally results in thin devices, typically in the range from 2 to 15 µm, the achievable minimal thickness of silicon-based probes is usually limited to 15–20 µm using silicon on insulator wafers (Lecomte et al. 2018; Wellman et al. 2019). Promising here is a recently presented method allowing localized removal of the complete silicon substrate, achieving ultra-thin probe tips of 2 µm thickness, only consisting of the metallization and silicon-oxide insulation layers (Otte et al. 2020). So, on the one hand, flexible microtechnology has the practical benefit of providing the fabrication methods to realize thinner devices, which will not fracture as easily even when made extremely thin. On the other hand, minimization of the width is usually achieved by resorting to established CMOS processes. These are limited to silicon substrates but can integrate high electrode numbers on shanks with a minimal cross-section (Steinmetz et al. 2021). The fact that Jun et al. (2017) demonstrated stable recordings over 2 months using a rigid Neuropixels array (Jun et al. 2017) emphasizes that the small scale enabled by CMOS silicon processing (70 µm × 20 µm in cross-section) may compensate the rigid mechanics. Implants that include CMOS technology, but with the silicon thinned down to where the device becomes flexible and embedded in a polymer substrate to facilitate handling, already exist. They represent an exciting future perspective to achieve flexible and small electrodes without sacrificing electrode integration density (Chiang et al. 2020; Moazeni et al. 2021).

**Fig. 2:**
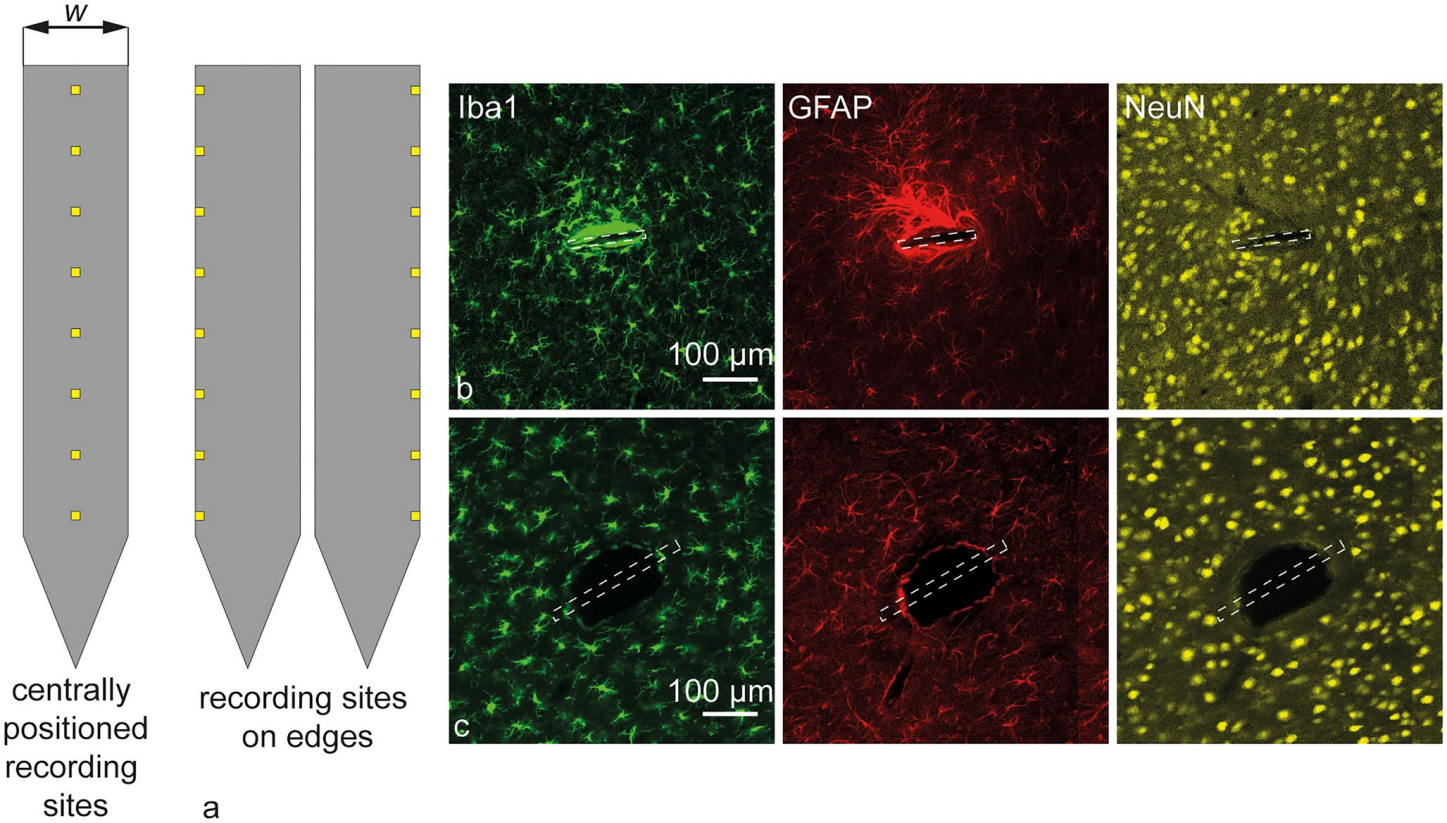
Scar shapes and their influence on the optimal positioning of recording sites: illustration (**a**) showing that recording sites can be oriented centrally (left) or along the edges (right) of planar probe shanks. Regarding the histology (**b**) (Rat049L, narrow device, 2 months) and (**c**) (Rat038R, wide device, 6 months), and results presented by Lee et al. (2018) to the implantation of narrow (*w* = 132 µm) and wide (*w* = 249 µm) devices, it becomes apparent that the wider the device, the more recording quality can be increased if sites are positioned on the edges. **b**, **c** Reproduced with permission from Lee et al. (2018)

If the integration of CMOS processes is not an option to narrow the probe, placing recording sites along the edges, rather than the center of the probe, as shown in Fig. 2a, has been shown to improve the recording quality of the device (Lee et al. 2018; Fiáth et al. 2021). This improvement could be due to the typical oval shape of the scar formed around wider probe shanks (Fig. 2b and c). Given that probe thickness remains small relative to the width, this process offers a workaround allowing wider probes to form satisfactory connections to neurons near the edge as well.

## Implantation techniques, initial trauma, and chronic outcome

### Surgical techniques for penetrating probes

For the successful implantation of a probe into the brain, the individual optimal force must be identified. This force corresponds to the minimal insertion force required to break through the dura or pia mater but is ideally smaller than the buckling force of the device (Lecomte et al. 2018). Per Eq. 1, defining factors for the buckling force are probe geometry (*w* and *t*) and Young’s *E* modulus. If the buckling force of the device itself does not exceed the insertion force, implantation must be ensured by applying assistive measures (Fig. 1e–g). These can be coatings (Fig. 1 e and g) or the application of shuttles (Fig. 1f). A possible additional categorization is the longevity of probe stiffening, with temporary stiffening being constrained to the exterior of the tissue (Fig. 1e), depending on the retraction time of a shuttle (Fig. 1f), or the degradation process of a coating (Fig. 1g).

Stiffening the probe only outside of the brain has enabled shuttle-free insertion even with highly flexible shanks (Patel et al. 2015; Hirschberg et al. 2017; Shoffstall et al. 2018). The aim of this technique is a temporary stabilization of the long shaft. A potential method is embedding the shaft in a dissolvable block of material and leaving only a short section protruding. This protruding section should be short enough to ensure that the probe does penetrate but not deflect upon contact with the brain surface. By stepwise dissolution of the stabilizing material, as shown in Fig. 1e, more of the probe becomes exposed so that it can be advanced one section at a time (Patel et al. 2015; Hirschberg et al. 2017). Shoffstall et al. (2018) presented a similar implantation method inspired by the mosquito’s labium: utilizing an insertion guide to stabilize a flexible probe only on the surface of the brain (Shoffstall et al. 2018). In both cases, the supporting stabilization does not enter the brain but remains outside of the tissue. By surrounding the exterior portion of the probe, it allows insertion without buckling.

A common short-term assisting technology for flexible insertion is to use a stiff shuttle (Fig. 1f) attached to the probe. The shuttle and probe are inserted together, as shown in Fig. 1f’-I and after implantation, the stiff shuttle is retracted (Fig. 1f’-II and f’-III). Depending on the mounting method used, retraction can occur immediately for mechanical fixation (Boehler et al. 2017; Luan et al. 2017; Kim et al. 2018) or as soon as the applied adhesive has dissolved (Kozai and Kipke 2009; Felix et al. 2012; Sohal et al. 2014; Du et al. 2017; Barz et al. 2020). Injectable probes (Liu et al. 2015) as the mesh of electrodes presented by Zhou et al. (2017) are a notable example for adhesion-free mounting to shuttles (Zhou et al. 2017). In this technique the shuttle consists of a syringe containing the structure to be implanted (Zhou et al. 2017). Depending on the desired implantation depth, the syringe is used to puncture the dura mater or to reach deeper into the tissue before releasing the electrodes.

As an alternative to the application of shuttles, a stiffening coating, as shown in Fig. 1g, can be used, which degrades over time after the probe is implanted (Fig. 1g’). Possible biodegradable or bioresorbable coating materials include polyethylene glycol (Lecomte et al. 2015; Guan et al. 2019), silk (Tien et al. 2013; Lecomte et al. 2015), poly(lactic-co-glycolic acid) (Pas et al. 2018), and dextran (Kil et al. 2019) amongst others (Lewitus et al. 2011; Gilgunn et al. 2012; Xue et al. 2018; Apollo et al. 2020). By adapting the material and structure of the coating, the resorption time can be tuned to the surgeon’s requirements. While intuitively, shorter degradation times seem favorable in terms of wound healing, longer time-frames offer the possibility of simultaneous controlled drug release, such as anticoagulants or anti-inflammatory agents (Lewitus et al. 2011; Jorfi et al. 2015; Lecomte et al. 2015; Wellman et al. 2018). Takeuchi et al. (2005) inverted this principle by fabricating a flexible probe with an integrated microchannel filled with PEG to stiffen the probe for implantation. After resorption of the PEG, this channel can be utilized for localized drug injections, presenting an additional possibility of modulating the implant microenvironment, e.g., to reduce inflammation and neurocoagulation (Takeuchi et al. 2005).

For all stiffening methods, one needs to differentiate between the cross-section of the device itself (remaining in the brain) and the implantation footprint, as shown in Fig. 1f’ and g’. The latter will here be used to describe the complete cross-section of the initial stab wound, including any additional material temporarily needed to support the probe during insertion. Consequently, while rigid probes typically have an implantation footprint equal to the cross-section of the shank itself, implantation of flexible probes usually requires the footprint to be larger than the probe itself. Keeping the coating thin reduces the implantation footprint, but as a trade-off, drug delivery capabilities and thus the possibility to influence immune responses remains limited. Notably, there is not always a big difference between the implantation footprint caused by a flexible device implanted by a shuttle (Fig. 1f) and rigid devices (Fig. 1b’) compared to a stiff one. This similarity is due to the fact that flexible probes are typically extremely thin (~ 10 µm thick), so the shuttle will contribute significantly to the thickness. As this can be realized by the same technology used for the microfabrication of silicon shanks, the implantation footprint may, in practice, be quite similar for the two categories of devices.

### Minimizing probe insertion trauma

When a probe is inserted into the brain, this action invariably inflicts trauma on the surrounding tissue. Multiple factors contribute to this initial trauma, including the purely mechanical damage to the brain structure and cells (Eles and Kozai 2020), any bleeding that occurs as a result of this (Kozai et al. 2012), and the impact of this bleeding in the surrounding neural tissue (Welle et al. 2020). As the probe advances through the tissue, it will destroy or displace cells and axons in its path, like a stab wound (Polikov et al. 2005; Michelson et al. 2018; Eles and Kozai 2020; Welle et al. 2020). The extent of this initial trauma both depends on the design of the device itself (cross-section and tip shape), the surgical technique, and the tools used to assist the insertion as summarized in the previous section (Sharp et al. 2009; Weltman et al. 2016; Lecomte et al. 2018; Wellman et al. 2018). In addition to the mechanical damage along the insertion path, any minor lateral vibration during insertion will induce a cutting motion, extending the implantation trauma beyond the actual dimensions of the device. For this reason, purely manual insertion should be avoided. A high-quality micro-drive that controls the lowering of the probes into the brain, minimizing the potential contribution of vibrations, is a straightforward first step towards reducing initial trauma.

If stiffening is necessary to allow implantation, it is beneficial if the stabilizing material can be limited to the area external to the brain, minimizing the implantation footprint (Apollo et al. 2020). As this calls for more complex implantation methods (Fig. 1e), the necessity of minimizing the footprint should be weighed against two main risks. First, minimizing the implantation footprint increases the risk of failure during surgery. Second, a less robust implantation method may result in longer surgeries, raising the possibility of bleeding or other complications. Moreover, Biran et al. (2005) found the stab wound itself is less problematic than the implant remaining in the brain (Biran et al. 2005). The work of Boehler et al. (2017) further supported this result, using a relatively large insertion shuttle (125 µm in diameter) to implant a 12-µm-thin flexible probe (Boehler et al. 2017). The short and long-term histology still demonstrated minimal gliosis, suggesting that a larger implantation footprint is tolerated well as long as the shuttle is immediately removed once the flexible device is in place.

When using a stiffening coating, the increased footprint may persist much longer than for a shuttle. In addition, the stiffening coatings usually are applied at thicknesses vastly exceeding that of the actual probe for sufficient mechanical stabilization. Thus, the rate at which this additional material resorbed may significantly influence whether the tissue will behave as favorably as following a shuttle insertion, or if this enlarged implantation trauma will negatively affect chronic-stage tissue integration. Notably, soft coatings have been reported to improve recording stability, probably due to a reduction in mechanical stress inflicted on the brain by the probe-brain micro-motions (Sridharan et al. 2019; Sridharan and Muthuswamy 2021). Furthermore, the presence of a coating alone has in other work been found not to significantly influence the immune reaction to the implanted probe (Lee et al. 2017). Specifically, promising results were presented by Kil et al. (2019) using Dextran as a resorbable stiffening coating (Kil et al. 2019). Sufficient stiffness for implantation was accomplished by a probe coating that added 37 µm to the implantation footprint. Their histological results after four months show that, after the coating got resorbed, tissue, including viable neurons, was able to infiltrate the region surrounding the probe.

In summary, insertion trauma is comparable to a stab wound, with an initial footprint, depending on the size of the probe plus any additional materials that will enter the brain to allow the implant to penetrate the tissue. This footprint should stay minimal, which can be accomplished, e.g., by minimizing vibrations during insertion using appropriate surgical equipment and mounting (Muthuswamy et al. 2005). However, data indicate that a small initial footprint may be less critical for tissue integration than the chronic properties of the probe, such as cross-section and overall mechanical properties (Biran et al. 2005; Boehler et al. 2017). Furthermore, when flexible probes are used, it might be favorable to resort to the most robust implantation methods, e.g., shuttles, to facilitate surgery and ensure reliable probe navigation in the tissue. One promising future possibility is engineering materials that are initially rigid but become compliant and flexible after implantation, avoiding the additional footprint of shuttles and coatings altogether (Nguyen et al. 2014).

### Insertion speed and dimpling

An additional contribution to mechanical trauma during implantation is “dimpling” of the brain surface, referring to the temporary compression of the most superficial layers of the brain. Dimpling is the result of the viscoelastic properties of the brain distributing the force imposed by the probe tip over a larger area. This temporal compression can damage tissue located further away from the insertion site (Bjornsson et al. 2006). While the amount of vascular damage inflicted by the implantation procedure is reportedly independent of the probe tip geometry (Bjornsson et al. 2006), the extent of dimpling depends on three main factors: the sharpness of the tip, the number of shanks implanted in parallel, and the speed at which probes are driven into the tissue (Rousche and Normann 1992; Patel et al. 2015; Boergens et al. 2020; Obaid et al. 2020). Typically, dimpling is less prominent with single shanks if the probe’s tip or shuttle are sharpened but contributes significantly to implantation trauma seen with multi-shank and bed-of-needle arrays (Schwarz et al. 2014). Insertion is commonly performed at high speed to circumvent substantial dimpling with such arrays, as this technique has proven efficient in reducing dimpling as a contributor to the initial trauma (Rousche and Normann 1992; Johnson et al. 2007). However, as slow insertion of probes can improve acute recording qualities (Fiáth et al. 2019), optimal insertion speed might differ between penetration and subsequent movement to reach deeper tissue layers (Bjornsson et al. 2006). For instance, Fiath et al. (2021) used insertion speeds of 2–5 µm/s for a comparative analysis with a range of silicon-based shank in rats (Fiáth et al. 2021). Notably, Cody et al. used ~ 16 µm/s for inserting the Utah Electrode Array in rats (Cody et al. 2018), which is far from the pneumatic insertion where the complete Utah Electrode Array is implanted in a millisecond (Rousche and Normann 1992, 1998). Interestingly, vibrational lateral movements of the probe seem to facilitate penetration of the dura mater and pia mater. Movements induced by ultrasonication can significantly reduce the insertion forces and thus help to prevent both brain dimpling and probe buckling (Chen and Lal 2015).

### Acute and chronic bleeding

**Fig. 3:**
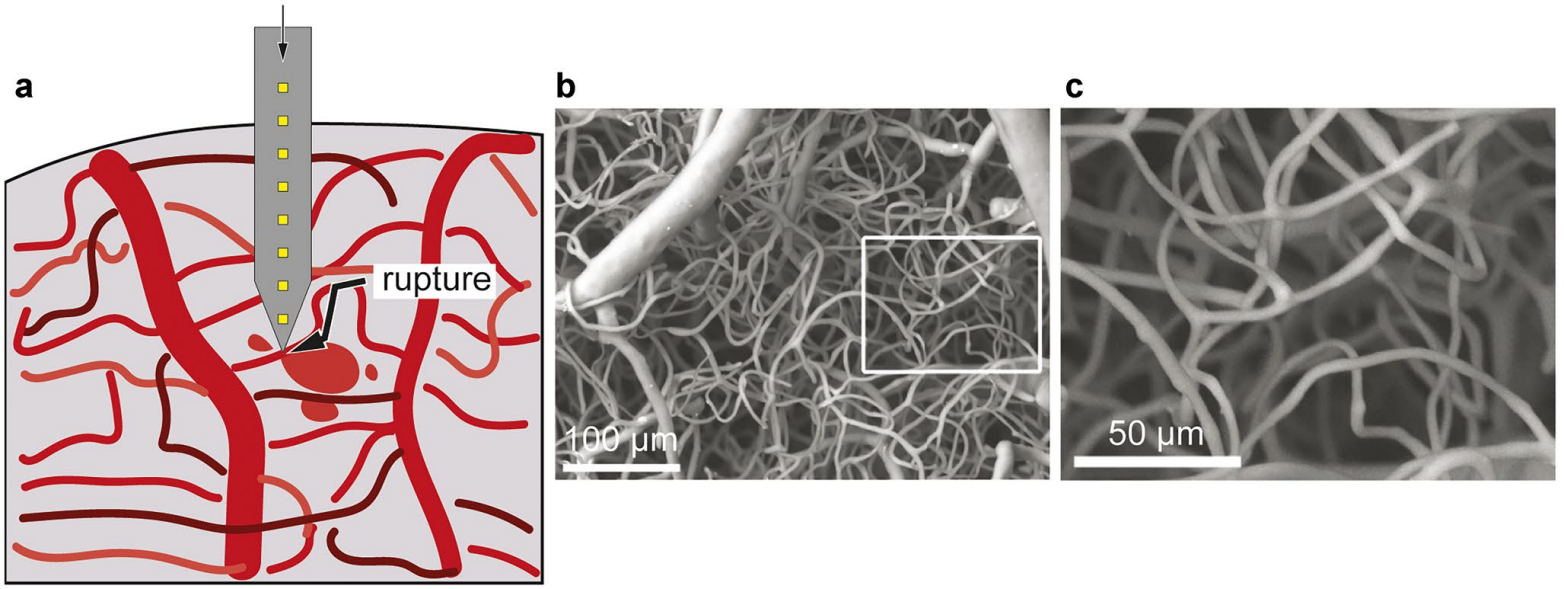
Illustration of a probe tip rupturing a blood vessel during insertion, causing bleeding (**a**). Scanning electron micrographs (**b**) and magnification of the inset (**c**) of the dense cortical vascularization in the brain got obtained by Wälchli et al. (2017). The corrosion casts shown are from P10 WT mice. **b**, **c** Reproduced with permission from Wälchli et al. (2017)

Aside from the mechanical damage, a direct consequence of insertion is bleeding (Fig. 3a), constituting a localized trauma itself. When major bleeding occurs, localized fluid accumulation may increase pressure around the disrupted vessel (Polikov et al. 2005). Similar to the previously described tissue compression caused by dimpling, tissue compression and increased cranial pressure caused by bleeding, can further harm the surrounding neurons and induce immune responses. Dysfunctional blood supply is a contributing factor in neurodegenerative disorders (Sweeney et al. 2018). Thus, changes to the brain vasculature caused by implantations might additionally affect overall normal brain functioning. If a vessel supplying two brain regions is breached, this can cause a decrease in blood flow rate in both regions essentially leading to depletion from proper blood supply (Cox et al. 1993; Woolsey et al. 1996; Bjornsson et al. 2006). In the worst case, this could create stroke-like effects even for areas further away from the initial insertion side and not directly affected by bleeding or dimpling during implantation. Notably, the diameter of arteries and thus the risk of extensive damage, decreases with cortical depth. Therefore, it is essential to avoid the region around the surface vessels during implantation to prevent major bleeding and subsequent negative effects (Kozai et al. 2010). Nolta et al. (2015) reported that rupture of larger vessels combined with astrogliosis and loss of tissue correlated with declining recording qualities (Nolta et al. 2015). Kozai et al. (2010) performed quantitative analysis on the short- and long-term outcomes of insertions rupturing a larger (arterial insertion) versus a smaller vessel (capillary insertion) (Kozai et al. 2010). Arterial insertion resulted in a reduction in both recording yield immediately and signal-to-noise ratio when evaluated after 7 weeks. Even when major bleeding is avoided with the appropriate surgical techniques, it is close to impossible to penetrate the brain tissue without inflicting any vascular damage given the dense vascularization of the brain (Ohtake et al. 2004), as is clear from the he corrosion casts shown in Fig. 3b and c, generated from 10-day-old (P10) wild-type (WT) mice (Wälchli et al. 2017). In their recent work, Wälchli et al. (2021) showed that vascular density increases from postnatal to adult mouse brains (Wälchli et al. 2021).

In the last decade, it has become increasingly clear that a leaky BBI or “microbleeding” may continue long after the initial implantation. Such microbleeding can on its own be a driver for inflammation, glial scar formation, and neuronal cell death—both because of the infiltration of blood-borne cells, such as macrophages (Ravikumar et al. 2014), coagulation factors, such as fibrinogen (Schachtrup et al. 2010), and macromolecules inducing neural excitation, promoting further BBI alteration (Potter et al. 2012; Saxena et al. 2013). This correlates with the observation that blood along the probe tract induces stronger acidosis, an expected consequence of more severe local inflammation (Johnson et al., 2007). In a recent study, Welle et al. (2020) combined in vivo two-photon imaging of neurons with optical coherence tomography to allow label-free imaging of blood vessels (Welle et al. 2020). They studied the interplay between vascular and structural dynamics around single- and multi-shank silicon probes over three months. This multimodal imaging allowed both acute and progressive alterations to be examined and revealed an intriguing interplay between intermittent episodes of blood flow decrease that resulted in local hypoxia, which in turn correlates with neuronal atrophy. The mechanisms behind these hypoxic events are not entirely clarified. However, the authors proposed that the cause was the mechanical interplay between the stiff shanks and the pulsating softer tissue (Welle et al. 2020).

### Summary of how to minimize implantation trauma

In conclusion, three main points must be considered to avoid pressure increases during implantation and minimize the initial trauma:

1. Dimpling of the dura is to be avoided.
2. Blood vessels should not be damaged.
3. The displacement of tissue is to be minimized.

However, these three criteria seem to contradict each other. Sharpening the tip of a probe to prevent dimpling increases the risk of vessel injury. If blunt probe tips are chosen to avoid cutting vessels during implantation, they will inevitably push aside vasculature and compress neural tissue during penetration. Downsizing the probe footprint can minimize the displacement of tissue but will make the implantation more difficult. Previous studies point towards small, cylindrical probes being advantageous in avoiding BBI breach (Karumbaiah et al. 2013). However, the optimal combination of probe shape, dimensions, material, and implantation method, addressing all three points to avoid localized pressure increase in the brain, is still unknown.

## Cells contributing to the probe tissue interface

During the last years, the understanding of tissue response at the cellular level has increased in complexity. The following brief overview of the most relevant cells reacting to neural probe implantations is ordered chronologically according to the recognition of their significance for this reaction.

### Astrocytes and reactive gliosis

The inflammation and reactive gliosis that leads to the formation of a glial scar was recognized early as one of the main challenges to overcome for neural interfaces to be long-term stable (Polikov et al. 2005; Tresco and Winslow 2011; Sridharan et al. 2013). The scar is formed as astrocytes become reactive in response to the foreign object, the implant, creating a tight encapsulating sheet, efficiently walling off the “intruder” from the brain. Thus, the scar constitutes a structural and biochemical barrier, separating the electrodes from the signaling tissue, attenuating the signals, and reducing the contact quality. Guttenplan et al. (2021) report on mechanisms of neural cell death caused by reactive astrocytes in neurodegenerative diseases. Similar effects might occur around implants, in the case of astrocytes staying chronically active and accumulating in neural scar tissue. Reactive astrocytes triggered by fibrinogen, a blood protein leaking into the neural tissue upon blood vessel damage during implantation, initiate scar formation (Schachtrup et al. 2010). The scar can be visualized by staining for cells expressing glial fibrillary acidic protein (GFAP) prominently expressed by the reactive astrocytes. Electrochemical impedance spectroscopy has been used in vivo to quantify the impedance of the insulating astrocytic layer (Williams et al. 2007). Typically, as more extensive reactions are associated with increased in vivo impedance, the initial primary hypothesis was that the decline in recording performance overtime was the direct consequence of the insulating properties of this encapsulating sheath. As different forms of encapsulation layers result in characteristic in vivo impedance changes, the use of in vivo impedance spectroscopy has been proposed for monitoring the extent of gliosis surrounding electrodes by studying the gradual increase of impedance (Cody et al. 2018). Anticoagulant therapies specifically targeting fibrinogen have shown a reduction in, but not a prevention of scar formation (Schachtrup et al. 2010).

### Neurons and neurodegeneration

In addition to the direct insulating properties of the gliotic scar, neuronal cell death has been identified as a fundamental reason for the loss of signals (Biran et al. 2005; McConnell et al. 2009). The most common histological marker of neurons surrounding the implant is NeuN. It is sometimes complemented by a neurofilament label such as MAP2. The distance between silicon shanks and first healthy neurons can exceed several hundred µm within 4–8 weeks (Biran et al. 2005). By comparing the loss of neurons surrounding a stab wound to that surrounding a probe, Biran et al. (2005) could also differentiate between the loss of neurons directly caused by the initial implantation trauma and the continued degeneration that is related to the presence of the implant over weeks to months (Biran et al. 2005). In a more recent study, Wellman et al. (2019) implanted 15-µm-thick silicon shanks with a tapered width from 123 µm to 33 µm (Wellman et al. 2019). The device’s presence over 28 days impacted neuronal density and axonal filaments beyond 150 µm (Wellman et al. 2019). Signs of neuronal apoptosis were most prominent closer to the device (Wellman et al. 2019). As the expected radius around electrodes for efficient recording of units is < 150 µm (Buzsaki 2004), much effort  has been invested in understanding the underlying causes of neural loss to find strategies to avoid this shortcoming. Pharmacological treatment to suppress the initial immune reaction has, this far, not resolved the challenge to prevent recording quality degradation over time. In summary, the biggest problem to solve is not the acute but rather the chronic trauma and immune reaction (Gaire et al. 2018).

It is worth noting that labeling neurons close to the device does not always correlate with good signal recordings (Kozai et al. 2014; McCreery et al. 2016; Michelson et al. 2018). Similarly, according to recent studies, reducing the insulating properties of the scar also appears non-critical to the recording function (Purcell et al. 2009; Malaga et al. 2016). Michelson et al. (2018) indicated that many studies report a poor correlation between recording quality and histological findings, stating histology without functional interface evaluation needs cautious interpretation (Michelson et al. 2018). Neurons staining positively for NeuN still could be functionally impaired, indicating that strategies should not focus on preserving NeuN positive cells alone but also the native function of the local neural network. Recent work by Welle et al. (2020) revealed more details on neuronal loss and correlated neuronal atrophy with local hypoxic events (see previous section) (Welle et al. 2020). In their study combining two-photon imaging with implanted silicon shanks, the authors confirmed that device implantation, in addition to the initial mechanical damage, resulted in progressive loss of neuronal dendrites over months. They report both a loss of dendrite density and reduced “overall branching and process complexity,” extending several hundred micrometers away from the interface (Welle et al. 2020). The role of secondary damage caused by denervation of remote healthy neurons, as a result of neuronal cell death close to the implant has not been addressed so far (Willems et al. 2016; Vlachos et al. 2013).

### Microglia, macrophages, and neuroinflammation

Besides hypoxia, one possible cause of neuronal loss is extensive inflammation. This effect can create a neurotoxic microenvironment surrounding the implant. Inflammation, mediated by reactive microglia, is expected in the acute phase following insertion but could also occur during the continued chronic response. In healthy brains, various physiological functions implicate microglia (Prinz et al. 2021), considered “first-responders” to brain damage and pathogens as the brain’s resident immune cells (Kawabori and Yenari 2015). Histologically, reactive microglia are typically labeled using IBA-1 (alternatively ED-1) and, for implanted probes, IBA-1 positive cells are part of the chronic inflammation in the gliotic region (Kozai et al. 2012; Potter et al. 2012). The microglial response to an implant is thereby different from that of a stab wound. While both events result in cell death, in the latter case, the initial microglial response typically fades away after some time, e.g., in the study by Potter et al. (2012), microglia returned to baseline within 16 weeks (Potter et al. 2012). Recently developed tools for the visualization of microglia in living tissue (Masuda et al. 2020) will greatly facilitate a spatiotemporal analysis of microglia dynamics.

Microglia fulfill their role as defenders of the brain and clear away introducers by phagocytosis or by releasing inflammatory and cytotoxic mediators. Two-photon microscopy imaging studies of probes in the brain have shown that microglia are recruited and activated immediately at implantation (Kozai et al. 2012, 2014). As implants cannot be phagocytized, it is reasonable that they induce a continuous release of pro-inflammatory cytokines in migroglia, resulting in a chronic inflammatory state (Potter et al. 2012). Histology around implanted silicon shanks shows an accumulation of reactive microglia (Biran et al. 2005; Wellman et al. 2018) and an increased secretion of pro-inflammatory cytokines, potentially affecting cell viability (Edell et al. 1992; Biran et al. 2005). Inflammation might not be the only reason for the neuronal loss, suggested by the data from Welle et al. (2020). Peak inflammation was expected at four weeks, whereas the negative impact on dendrite structures continued over 3 months (Welle et al. 2020). Hypoxia caused by increased pressure and limited perfusion (Welle et al. 2020) or neurotoxic factors derived from astrocytes (Guttenplan et al. 2021) could be additional causes for neural death.

Activated microglia can cause neurodegeneration. However, they are also key players in brain homeostasis and repair mechanisms and can exert neuroprotective effects by suppressing rather than promoting inflammation (Cherry et al. 2014). Prasad et al. (2014) reported that their observed recording performance did not correlate with microglial activation, while vascular injury and bleeding always led to a decline in recording quality (Prasad et al. 2014). Hence, blood-derived cells, such as circulating monocytes, macrophages, and other blood components entering the brain after injury, are most likely contributing significantly to neuroinflammation.

Microglia are not “dangerous” cells around implanted microdevices but show heterogenic and possibly neuroprotective properties (Prinz et al. 2021), and  cannot alone explain why long-term neurodegeneration continues well after the expected inflammation peak. In addition, Salatino et al. (2017) suggested glial cells could even contribute to therapeutic effects of electrical stimulation, and this interplay should be investigated further (Salatino et al. 2017). Unfortunately, most studies did not differentiate between microglia and blood-derived monocytes and macrophages (Ravikumar et al. 2014). It appears that BBI dysfunction is a significantly more severe issue than the accumulation of microglia cells and persisting neural inflammation. More work is required to clarify the distinct roles of different microglia and macrophages at various stages after implantation.

### Oligodendrocytes and pericytes

Over the last few years, other cell types have been implicated as possible participants in the glial scarring process. In particular, oligodendrocytes and pericytes have been recognized as significant factors in maintaining brain-circuit function (Wellman et al. 2019, 2020). In their study using silicon shanks, Wellman et al. (2019) found a decrease in oligodendrocytes surrounding the device from day 7 to 28 following implantation (Wellman et al. 2019). Subsequently, the oligodendrocyte density was restored (Wellman et al. 2019). The authors pointed out that the presence of oligodendrocytes could play a role in the healing processes (Wellman et al. 2019), and that the importance of remyelination in the tissue surrounding the implant should be investigated further (Wellman et al. 2019). Chen et al. (2021) reported oligodendrocyte injury in the form of deformation after probe implantation and beginning of myelin injuries was observed 3 days later (Chen et al. 2021). Remyelination by oligodendrocytes is suppressed by fibrinogen entering the neural tissue upon BBI breach (Petersen et al. 2017) which suggests fibrinogen might be a reason for the following two effects: first, continuous degeneration in oligodendrocyte somas, and second, demyelination within a range of 100 µm surrounding an implantation site (Chen et al. 2021). Thus, therapeutical intervention for mitigation of fibrinogen might promote the remyelination of axons post-implantation (Petersen et al. 2017).

Pericytes, cells wrapping around the smaller brain capillaries, are essential contributors to sustaining the BBI function (Sweeney et al. 2016). Wellman et al. (2019) noted a drastic decrease in pericytes (determined by PDGFR-β staining), which correlated with increased BBI leakage over time (Wellman et al. 2019). The contribution of pericytes to the re-vascularization of damaged tissue, and their role in BBI dysfunction, may be of substantial importance in assessing the outcome of the glial scar formation. Future research should invest in carefully mapping, i.e., structurally, functionally, and molecularly characterizing the tissue surrounding penetrating neural probes (Sweeney et al. 2016, 2018).

## Summary/outlook

There has been remarkable progress in developing novel, tailored intracerebral probes and studying the immune response to their implantation. Nevertheless, some questions remain unanswered. Optimizing the functionality and implantability of neural probes while minimizing induced immune response requires further investigation. Moreover, studies on glial scarring typically focus on the reaction to single penetrating shanks, and clarification is needed to determine to what extent these results can be extrapolated to the multi-shank and bed-of-needle arrays required by most applications. Interdisciplinary teams consisting of neuroscientists, immunobiologists, and engineers must work closely to clarify implantation challenges (Kozai 2018). Probe implantability can be evaluated ex vivo or using finite elements (Subbaroyan et al. 2005) or mechanical models (Sommakia et al. 2014) to mimic brain and dura. Immunoreactivity, however, is a more complex aspect of neural probe integrity and can currently not be modeled ex vivo as not all mechanisms are fully understood (Sommakia et al. 2014). Chronic in vivo imaging has expanded the potential for longitudinal studies of the tissue reaction to probes, enabling more efficient analysis of the interaction of cells and vasculature (Kozai et al. 2012; Wellman et al. 2019; Welle et al. 2020). It is likely that such analysis, complemented with endpoint histology, will accelerate progress towards understanding the probe-tissue interface and ultimately permit the design of interfaces that minimize deleterious glial scarring.

Insertion of probes in the brain inflicts trauma, which will trigger a reaction of the brain immune system, resulting in the formation of a scar. The question of whether bleeding and scar formation is inevitable remains open for now. This review consolidates information needed to answer this question from an engineering perspective, focusing on mechanics, materials, surgical tools, and techniques. There is still no device that can be inserted without inducing any bleeding. Considering that small deposits of fibrinogen and other blood components can trigger gliosis even in the absence of a probe, a complete elimination of glial scarring will be challenging to achieve, relying solely on implant design. A pharmacological approach may in addition be needed to accomplish this goal. Strategies can be either reprogramming the biological reaction or completely removing or disguising the biological trigger. Relevant inspiration for this task could come from the stroke research community, where solutions to dissolve fibrinogen deposits have been investigated to reduce gliosis after stroke (Schachtrup et al. 2010; Liu et al. 2011). Improved understanding of the immune reaction and scar formation to different probes enables the application of increasingly specialized pharmacological treatments supporting probe-tissue integration. Nevertheless, the device application dictates the optimal approach to managing the immune response, bleeding, and regeneration. For certain clinical applications neural regeneration surrounding the implant may be sufficient, e.g., to allow a neuroprosthetic brain-machine interface to be controlled from the motor cortex (Courtine et al. 2013). Nevertheless, damage prevention is of the utmost importance and should be a main priority in device design. A less disruptive interface keeping the surrounding neural circuitry intact would greatly improve the possibilities to access high quality electrophysiological data both for neuroscience and therapy.

Today, the best implants are those that are customized to their intended application. For example, if the aim is high-resolution recordings from a specifically targeted region, CMOS probes are the method of choice. In contrast, mesh probes are preferable if the location for recordings is not precise, but the immune reaction should be minimized. The final selection of the probe is always a compromise between functionality, implantability, and biocompatibility. Hybrid, floating probes might be the most generally applicable current solution. Most importantly, much evidence indicates that combining optimal surgical methods with flexible probes and/or minimized cross-section of the single shanks can ameliorate glial scar formation. This improvement seems sufficient for long-term, stable, functional interfacing of neurons even when the implant is not entirely scar-free.
